# Solvolyses of Benzoyl Chlorides in Weakly Nucleophilic Media

**DOI:** 10.3390/ijms12084805

**Published:** 2011-07-28

**Authors:** Thomas William Bentley, Haldon Carl Harris

**Affiliations:** Chemistry Unit, Grove Building, School of Medicine, Swansea University, Swansea SA2 8PP, Wales, UK; E-Mail: carl.harris1@googlemail.com

**Keywords:** solvolysis, substituent effects, solvent effects, acylium cations

## Abstract

Rate constants and activations parameters are reported for solvolyses of *p*-Z-substituted benzoyl chlorides (**1**, Z = OMe, Me, H, and Cl) in 97% w/w hexafluoroisopropanol-water (97H). Additional kinetic data are reported for solvolyses in acetic and formic acids. Plots of log *k vs*. σ_p_ in 97H are consistent with previous research showing that a cationic reaction channel is dominant, even for solvolyses of **1**, Z = NO_2_. A benzoyl cation intermediate was trapped by Friedel-Crafts reaction with 1,3,5-trimethoxybenzene in hexafluoroisopropanol. The results are explained by an S_N_2-S_N_1 spectrum of mechanisms with variations in nucleophilic solvent assistance. *Ab initio* calculations of heterolytic bond dissociation energies of various chloro- and fluoro-substituted and other benzoyl chlorides are correlated with log *k* for solvolyses.

## 1. Introduction

As well as the expected initial addition to the carbonyl group, solvolyses of carboxylic acid halides can occur via cationic processes in weakly nucleophilic solvents (e.g., fluorinated alcohols [1]). Cationic processes can also occur in relatively nucleophilic aqueous solvents, if electron-donating groups are present (e.g., in *p*-methoxybenzoyl chloride (**1**, Z = OMe) [2] or *p*-dimethylaminobenzoyl fluoride (**2**) [3]) or if nucleophilic attack at the carbonyl group is sufficiently sterically hindered (e.g., by 2,6-substituents in benzoyl chloride derivatives **3** [4,5] and **4** [6].

Correlation analysis based on the extended Grunwald-Winstein (GW) equation has played a major role in providing quantitative evidence for the simultaneous operation of both cationic and addition reaction channels for solvolyses of a single substrate (e.g., benzoyl chloride, **1**, Z = H) [2,7], 2,4-dichlorobenzoyl chloride (**5**) [6] and 2,6-difluorobenzoyl chloride (**6**) [8], as the polarity and/or nucleophilicity of the solvent is varied. Mechanistic changes also make benzoyl chlorides suitable as molecular probes for determining the polarity of the cavity of cyclodextrins [9].

The spectrum of mechanisms is well characterized only at the extremes. The carbonyl addition pathway is exemplified by *p*-nitrobenzoyl chloride (**1**, Z = NO_2_), and shows a high response to changes in solvent nucleophilicity, a low response to changes in solvent ionizing power, and a high solvent kinetic isotope effect [3,10].

The cationic reaction channel is best exemplified by solvolyses of **2**, which shows common ion rate depression in water, characteristic of an S_N_1 pathway via a “free” cation intermediate, even in a nucleophilic solvent [3]. Other substrates including **1**, Z = OMe [2,5] and carbamoyl chlorides (e.g., Ph_2_NCOCl [11]; Me_2_NCOCl [12]) show product ratios in mixed alcohol-water solvents, characteristic of reactions via solvent separated ion pairs.

The main purpose of the research now reported was to obtain further information about the cationic reaction channel by studying solvolyses of *p*-substituted benzoyl chlorides (**1**) in weakly nucleophilic solvents. The data lead to an analysis of both solvent and substituent effects. Also included are experiments designed to trap cationic intermediates, and *ab initio* calculations of substituent effects.

## 2. Results and Discussion

### 2.1. Reliability of Rate Constants

The rapid response conductimetric method was employed, collecting data at preset times and storing the readings in a digital voltmeter before processing [10]. In general, fluorinated alcohols containing small amount of added water perform well in conductimetric studies, but substrates must be dissolved rapidly. Typically, reactions were initiated by injecting a few μL of a 1% solution of substrate in dry acetone into *ca*. 3 mL of solvent, so substrate concentrations are <10^−3^ M. Consistent trends in activation parameters were observed (Table 1). Some of the results for solvolyses in 97% w/w hexafluoroisopropanol-water (97H, Table 1) required extrapolations from data at lower temperatures to 25 °C. For solvolyses of **1**, Z = H, the extrapolated value is *ca*. 5% lower than our previous measurement [13], which may be due to small variations in solvent batches.

For solvolyses of **1**, Z = Cl in 97H (Table 1) continuous monitoring of changes in absorbance in a thermostatted UV cell at 25 °C led to rate constants in good agreement with the conductimetric result; this establishes the validity of the UV spectrophotometric method, which we had not previously used for acid chlorides. However, our data are *ca*. 40% greater than the previously published value, obtained titrimetrically [7]. Titrimetric analyses require higher substrate concentrations, and as shown below spectrophotometric and titrimetric methods may be more susceptible to problems of dissolving the substrate.

For formolyses, the conductimetric method was checked by investigating solvolyses of *t*-butyl chloride, which gave *k* = (1.07 ± 0.07) × 10^−3^ s^−1^, in agreement with the literature value of 1.05 × 10^−3^ [14]. Formolyses of benzoyl chloride (**1**, Z = H) were initiated either by injecting neat substrate or a 10% solution in dry acetone, and the latter gave *ca*. 5% higher values, indicating that dissolution rates may be contributing. For formolyses of **1**, Z = Cl, we employed both conductimetric and UV spectrophotometric methods (Tables 2 and 3). Rate constants obtained from UV data were significantly lower than the conductimetric results; the differences may be due to problems dissolving the substrate, although ultrasonication prior to conductimetric measurements and injections with a spring-loaded syringe into the UV cell did not lead to significantly different results.

Acetolyses are typically investigated titrimetrically in the presence of carboxylate buffer [14], but under these conditions reactions of benzoyl chloride (**1**, Z = H) were very rapid. Presumably there is base catalysis, unlike formolyses of **1**, Z = H which have been found not to be catalysed by added formate [16]. In the absence of buffer, acetolyses did not go to completion, and our value is based on monitoring <50% reaction is significantly lower than the published value (Table 3, footnote *d*). For acetolyses of of **1**, Z = OMe, the UV method gave higher rate constants than the titrimetric results, unless acetic anhydride was added (Table 3, footnote d). Monitoring the reaction by HPLC as it proceeded showed that there was an unstable intermediate product, presumably the mixed anhydride which reacts with traces of water to give 4-methoxybenzoic acid (the only product detected by HPLC after long reaction times). Titrimetric data require *ca*. 50-fold higher concentrations of substrate than UV or HPLC, so traces of water could be removed in the early stages of the reaction.

### 2.2. Kinetic Data (Substituent Effects)

Plots of log *k vs.* a substituent parameter such as σ [16,17] or σ^+^ [1,3,18], depend on the solvent and substituent. In 50% acetone-water [17] and 50% ethanol-water [18], the plots are U-shaped; the carbonyl addition pathway is aided by electron-withdrawing substituents (positive slope), whereas the cationic pathway is aided electron-donating substituents (negative slope).

Our results (Figure 2) refer to solvents of greater ionizing power and/or lower nucleophilicity, so the cationic pathway is usually (but not always) dominant; the plots are approximately linear but slopes vary significantly. Logarithms of rate constants in 97H, plotted *vs*. both σ_p_ and σ_p_ ^+^ (Figure 2a), show the unexpected result that the data fit σ_p_ better than σ_p_ ^+^. Data for **1**, Z = NO_2_ fit the plot, whereas it was excluded from the correlation line using the GW equation for the addition reaction channel because it reacted significantly faster than predicted [7]. These results can be explained consistently if it is assumed that the cationic reaction channel is now dominant, even for solvolyses of **1**, Z = NO_2_.

**Figure 1.**
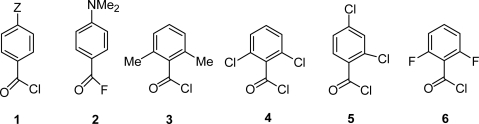
Halides (**1**–**7**) are named as follows: (**1**) *p*-Z-substituted benzoyl chorides; (**2**) *p*-dimethyaminobenzoyl fluoride; (**3**) 2,6-dimethylbenzoyl chloride; (**4**) 2,6-dichlorobenzoyl chloride; (**5**) 2,4-dichlorobenzoyl chloride; (**6**) 2,6-difluorobenzoyl chloride.

**Figure 2.**
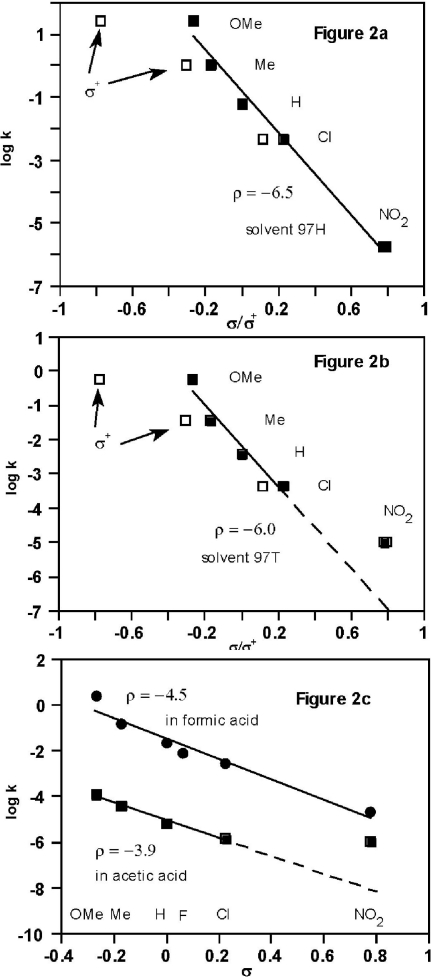
Plots of logarithms of rate constants for solvolyses in various solvents *vs*. σ and σ^+^ (substituent parameters from reference [19]): (**a**) solvent 97% hexafluoroisopropanol-water (97H), ρ = −6.5 ± 0.5, data from Table 1; (**b**) solvent 97% trifluoroethanol-water (97T), ρ = −6.0 ± 1.0, data from reference 1; (**c**) solvents acetic (ρ = −3.9 ± 0.3) and formic acids (ρ = −4.5 ± 0.5), data from Table 3.

A plot of log *k vs*. σ_p_ ^+^ for reactions in 97% w/w trifluoroethanol-water (97T) is close to linear (Figure 1 of reference [1], open squares in Figure 2b, σ_p_ = σ_p_ ^+^ for H and NO_2_), suggesting the operation of a cationic process throughout the series **1**, Z = OMe to NO_2_. However, rates of solvolyses of **1**, Z = NO_2_ in 97T fit the extended Grunwald-Winstein (GW) equation for the addition channel [7]. The new correlation line of log *k vs.* σ_p_ instead of σ_p_ ^+^ supports the alternative viewpoint [7] that **1**, Z = NO_2_ deviates from Figure 2b because of a change to the addition reaction channel.

A more complete account would be based on a dissection of substituent effects into resonance and non-resonance contributions, based on the Yukawa-Tsuno (Y-T) Equation 1 [20]; *r* = 1 corresponds to a σ*^+^* plot, so the results (Figure 2) indicate that resonance demand (*r*) is smaller for acylium ions than for cumyl cations (the reference substrates for σ*^+^*).

(1) $$\text{log}\;k/k_{0}=\rho [\sigma +r(\sigma^{+}-\sigma )]$$

Significantly, formolyses of **1**, Z = F are slower than for **1**, Z = H, in agreement with respective values of σ_p_ (+0.062 and 0.0) and in contrast to values of σ_p_ ^+^ (−0.073 and 0.0). A linear σ_p_ plot for formic acid is shown (Figure 2c), but as in Figure 2a,b, a curve could be drawn. The point for Z = OMe deviates most from a linear plot, and Equation 1 would remedy this.

More convincingly, the σ_p_ plot for acetolyses (Figure 2c) shows a large deviation for **1**, Z = NO_2_, whereas log *k* for acetolysis fits the GW equation [7] for the addition reaction channel. Consequently, these two independent types of correlation analysis support the assignment of acetolyses of **1**, Z = NO_2_ to the addition reaction channel. Acetolyses of **1**, Z = Cl may deviate slightly from Figure 2c, and the predictions of the published GW equations [7] are ambiguous: log *k* = −5.21 for the cationic channel and −6.45 for the addition channel [21,22], midway between the observed value (log *k* = −5.85, Table 3).

The following theoretical calculations provide some support for the choice of σ_p_ over σ_p_ ^+^, in the absence of sufficient data for Equation 1.

### 2.3. Theoretical Calculations of Substituent Effects

Results of correlation analysis (Figure 2) can be compared with expectations based on calculations for acylium ions in the gas phase. A recent reassessment of gas phase data for the benzoyl cation, supported by high level *ab initio* calculations [23], gives a value of 738.8 ± 3.3 kJ/mol for the heat of formation at 298 K; this gives a heterolytic bond dissociation energy (HBDE) for benzoyl chloride (**1**, Z = H) of 148 kcal/mol in satisfactory agreement with the calculated value of 150.1 kcal/mol [24].

Much lower level calculations give satisfactory results for substituent effects for hydride transfers in cumyl cations [20] and for transfer of chloride ion from a substituted benzoyl chloride to the benzoyl cation (Equation 2) [25], because the reactions are homodesmotic [26].
(2)
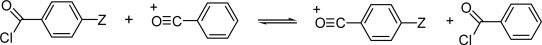


In contrast to expectations based on σ_p_ *^+^*, the results (Table 4) confirm that a *p*-fluoro substituent destabilises the benzoyl cation, as shown by a positive sign for the stabilisation energy (SE, Equation 2); interestingly, theoretical calculations also showed that a *p*-fluoro substituent destabilised cumyl cations in the gas phase [20]. Also, destabilisation by *p*-nitro is larger than the stabilisation by *p*-methoxy, again contrary to expectations based on σ_p_ *^+^* [19].

**Figure 3.**
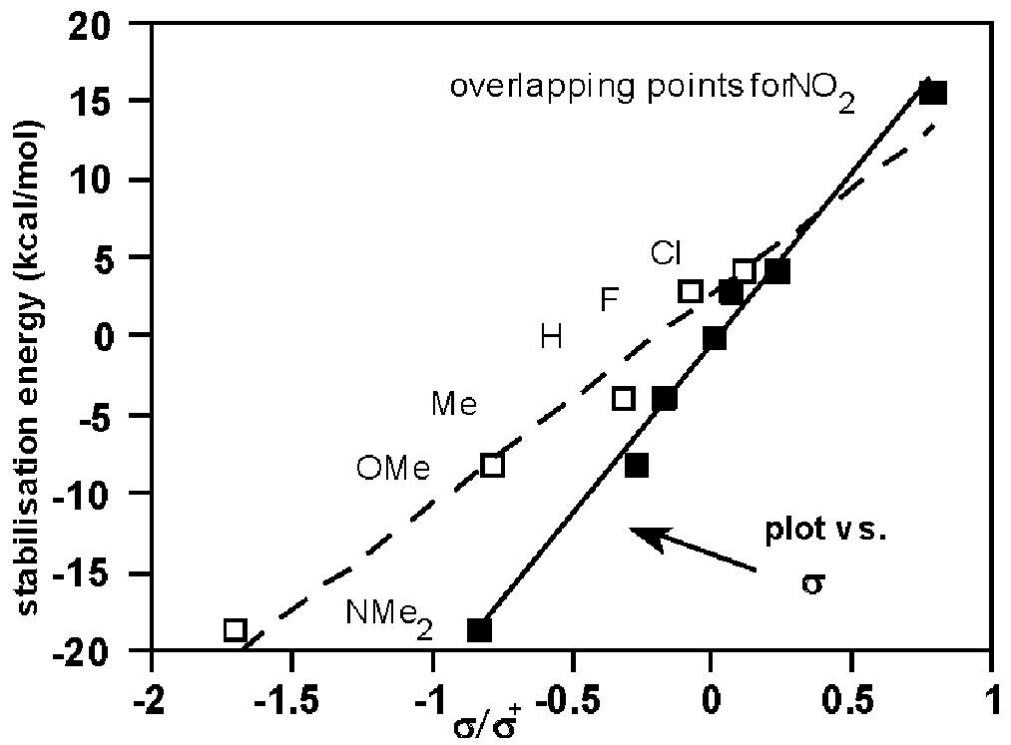
Correlations of HF/6–31G stablisation energies (Equation 2, Table 4) with σ_p_ and σ_p_^+^; slope: 21.7 ± 1.1; intercept: −0.56 ± 0.50; *r* = 0.994.

The correlation *vs*. σ_p_ (Figure 3) is good and includes the point for NMe_2_, so the range of HF/6–31G(d) stabilisation energies is 34 kcal/mol; the hatched line is a plot *vs*. σ_p_ ^+^ and shows significant deviations for H and NO_2_ (the open square symbols are hidden because σ_p_ = σ_p_ ^+^). The B3LYP data (Table 4) give a better correlation with σ_p_ ^+^ than σ_p_, and much more data fitted to the Y-T Equation 1 are needed to clarify the situation.

**Figure 4.**
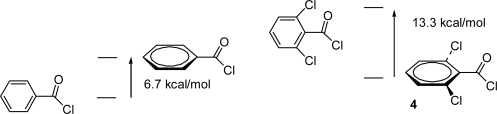
Preferred conformations of benzoyl chloride (planar) and 2,6-dichlorobenzoyl chloride (**4**, perpendicular); barriers to rotation are shown (from HF/6–31G(d) calculations); the higher energy species of each pair were characterized as transition structures, having one negative frequency.

The *ortho*-substituted compounds were calculated to be twisted out of plane, with a resulting loss of conjugation. The preferred conformations of 2,6-dichlorobenzoyl chloride (**4**) was calculated to be perpendicular (Figure 4). Conjugative effects are significant in planar neutral substrates (**1**), and electron donors (Z) elongate the C–Cl bond [26], so there must be a large increase in conjugation when an acylium ion is formed from a non-conjugated perpendicular conformation. Despite the presence of electron withdrawing halogen groups HBDEs for **4** are about the same as for the parent benzoyl chloride. The preferred conformation of the 2,6-dimethyl derivative (**3**) is close to perpendicular, and a high stabilisation energy is predicted (Table 4).

Extensions and improvements to previous work [24] on relating HBDEs to reactivity of seven acid chlorides in 97T at 25 °C can now be made. Equation 3 applies to five substrates (**1**, Z = OMe, Me, H, Cl and NO_2_) in 97H at 25 °C (Table 1), and is more precise than the one published earlier [24]; the error in the intercept is due to a long extrapolation, but the standard error in log *k* is only 0.074. Predictions based on Equation 3 are shown in Table 5.

(3) logk=(−0.318±0.004)×HBDE+46.4±0.7(n=5,  r=0.9997)

Hammett correlations could be applied to reactions of both 3,4-dichloro- and 3,5-dichlorobenzoyl chloride, and these also fit Equation 3 well (within log *k* = 0.4). All of the “experimental data” for remaining substrates (Table 5) required extrapolations (see footnotes). Reactions of *ortho*-substituted substrates are excluded from Hammett correlations, but Equation 3 is moderately successful and also provides useful insights. Substrates **3** and **4** have perpendicular conformations, and errors in predictions are about one order of magnitude in *k* (**3** is predicted to be slower and **4** is predicted to be faster than observed). The worst predictions are for **5** and **6**, and it may be significant that conformations of these two less sterically-hindered substrates are predicted to be twisted but not perpendicular; then the solvent may have a greater influence on the conformation and hence on changes in conjugation energy during ionization. There may also be steric hindrance on solvation [27].

### 2.4. Product Studies and Reaction Mechanisms

Reactions of benzoyl chloride with various nucleophiles were investigated in attempts to trap cationic intermediates. It is necessary to avoid the alternative situation where the nucleophilic “trap” induces a competing reaction pathway; this would lead to a rate enhancement predictable from the amount of new product (calc. RE), as observed in preliminary studies for amines in 97T at 25 °C (0.01 M *p*-nitroaniline gave a 5% calc. RE and 0.01 M *m*-nitroaniline gave a 60% calc. RE, in agreement with the observed RE).

**Figure 5.**
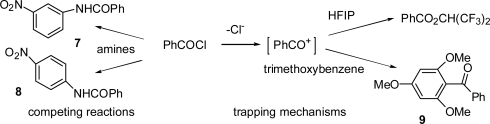
Added amines lead to competing reactions of PhCOCl to give anilides (**7** and **8**), but trapping of a PhCO^+^ intermediate may occur with 1,3,5-trimethoxybenzene to give (**9**).

Larger concentrations of weaker nucleophiles (electron rich aromatic substrates) were then investigated. Conductimetric studies of the reaction of benzoyl chloride (0.0002 M) in 97T containing methoxybenzene (0.2 M) showed rate retardation, and 4-methoxybenzophenone was not detected in the resulting solution (from HPLC analysis). Numerous other attempts using HPLC monitoring eventually led us to use much higher concentrations of benzoyl chloride (0.02 M) and 0.2 M 1,3,5-trimethoxybenzene as the trap. Under these conditions, dry trifluoroethanol gave only ester product and 97H gave mainly benzoic acid possibly with traces of 2,4,6-trimethoxybenzophenone (**9**). However, in 100% HFIP, the yield of **9** was estimated from HPLC and NMR to be approximately 50% (Figure 5), and a yellow solution was obtained; in this case it is reasonable to propose a trapping mechanism, assuming (but not demonstrating experimentally) the absence of a rate-product correlation.

In contrast to the difficulties in trapping the benzoyl cation, the 4-methoxybenzyl cation can be trapped by various arenes even in 97T [28]. Acylium ions are less likely to become “free”, and more likely to react at an earlier stage such as a solvent separated ion pair [29].

Correlation analysis plays a major role in mechanistic studies of acid chlorides, partly because other evidence such as stereochemistry or secondary deuterium kinetic isotope effects is much less readily applicable. The results of correlation analysis for weakly nucleophilic solvolysis media for substituent effects (Figure 2) and solvent effects [7] are consistent with a spectrum of mechanisms within a cationic reaction channel having S_N_2 character [13,30]; e.g., (i) ρ follows the order 97H > 97T > HCO_2_H > AcOH (Figure 2), as observed for solvolyses of secondary alkyl tosylates (Table VIII of reference [31]) for which an S_N_2-S_N_1 spectrum of mechanisms is more well established; (ii) when the extended Grunwald-Winstein equation is applied [7] to the cationic reaction channel of benzoyl chlorides (**1**), the parameter *l* (a measure of response to solvent nucleophilicity) increases from 0.31 for Z = OMe to 0.41 (Z = Me), 0.47 (Z = H), and 0.56 (Z = Cl).

A measure of the extent of nucleophilic solvent assistance (NSA) to heterolysis of the C–Cl bond is provided by comparisons of solvolyses of **1**, Z = OMe (assumed to react without nucleophilic solvent assistance (but see [32],) with other substrates; the rate ratio *k*_97H_/*k*_AcOH_ is 2.25 × 10^5^ for **1**, Z = OMe, and 8.9 × 10^3^ for **1**, Z = H, giving a minimum estimate of 25 for NSA. For comparison, acetolysis of cyclohexyl tosylate has an NSA of 28 [33], and nucleophilic attack is confirmed by the products (85% substitution with retention of stereochemistry in competition with 15% hydride shift [34]).

According to correlation analysis of solvent effects, solvolyses of acetyl chloride even in aqueous media fit the S_N_2-S_N_1 spectrum with a high *l* value of 0.8 [35,36], alternatively described [36] as ionization “with considerable nucleophilic solvation” [36]. Estimates based on kinetic data at 0 °C show that acetyl chloride reacts about 5 orders of magnitude faster than predicted from Equation 3, and a prior hydration mechanism could not be excluded [24,37]. Recent extensive DFT calculations included a wide range of acid chlorides and optimized structures of assemblies including molecules of water and acetone. Contrary to the dual channel mechanism, a single reaction channel having extensive transition state variation within a distorted tetrahedral geometry was predicted [38]. The acetyl cation was excluded as a possible intermediate in aqueous media, but a nucleophically solvated (weakly bonded covalently) acetyl cation *transition state* was not considered [38].

## 3. Experimental Section

The acid chlorides (**1**, Z = OMe, Me, H, F, and Cl) were commercial samples checked for purity by HPLC analysis of methanolysis products, and shown to contain <0.3% acid. Anilides (**7** and **8**, Figure 4) were prepared by reacting benzoyl chloride with the appropriate amine in methanol [39]. 4-Methoxybenzophenone was a commercial sample (Aldrich) and 2,4,6-trimethoxybenzophenone (**9**) was prepared by heating trimethoxybenzene, benzoyl chloride and zinc chloride in benzene under reflux for 4 hr. After workup, the crude product was recrystallised from methanol; m.p. 113−115 °C, lit: 115 °C [40]; ^1^H NMR (CDCl_3_): δ, 3.6 (6H, s); 3.8 (3H, s); 6.1 (2H, s); 7.3−7.9 (5H, m).

Solvents for kinetic studies were acetic acid (BDH Aristar), formic acid (BDH Analar 98–100%), and HFIP (distilled through a triple pass Widmer column). Kinetic methods, based on conductivity [1,10], spectrophotometric [31] and titrimetric [41] measurements, were as described previously.

HPLC methods were as described earlier [5,13], with UV detection at 270 nm. and elution typically with 60% methanol-water. Retention times (mins) were benzoic acid (1.4), anisole (6.0), trimethoxy-benzene (7.4), **9** (8.8), PhCO_2_CH_2_CF_3_ (10.9), 4-methoxybenzophenone (12.4), PhCO_2_CH (CF_3_)_2_ (17.0).

Calculations (Table 4) were performed using the standard Gaussian 03 [42] at the Rutherford Appleton laboratory on the Magellan service, using the EPSRC National Service for Computational Chemistry (NSCCS); all energies in Table 4 refer to structures having no negative frequencies.

## 4. Conclusions

Correlation analysis shows that substituent effects (using σ not σ^+^) and solvent effects (the extended Grunwald-Winstein equation) on solvolyses of benzoyl chlorides can be explained quantitatively by two competing reaction channels [7]. The independent correlations agree that solvolyses of **1**, Z = NO_2_ fit an addition reaction channel, except in hexafluoroisopropanol (HFIP).

The research reported above focused on the cationic pathway, favoured in weakly nucleophilic media and comparable with the S_N_2-S_N_1 spectrum for simple secondary tosylates [31]. Reactions are favoured by electron donating groups and values of ρ increase in the order AcOH < HCO_2_H < TFE < HFIP (Figure 2) due to decreases in nucleophilic solvent assistance. Product studies indicate that cationic intermediates cannot be trapped efficiently (except in HFIP (Figure 5)), consistent with acylium cationic transition states or intermediates encumbered by solvent acting as nucleophile.

## Figures and Tables

**Table 1.** Rate constants (*k*) and activation parameters for solvolyses of *p*-substituted benzoyl chlorides (**1**) in 97% w/w hexafluoroisopropanol-water (97H) *^a^*.

**Substrate *^b^***	**Temperature/°**C	***k*/s^−1^**	**ΔH^≠^/kcal mol^−1^**	**ΔS^≠^/cal mol^−1^ K^−1^**
**1**, Z = OMe	−20.15	(3.15 ± 0.03) × 10^−1^	14.2	−4.3
	−9.90	(9.87 ± 0.02) × 10^−1^		
	25.0 *^c^*	27		
**1**, Z = Me	−10.0 *^d^*	(4.08 ± 0.06) × 10^−2^	14.0	−11.4
	0.1	(1.14 ± 0.01) × 10^−1^		
	25.0 *^c^*	1.07		
**1**, Z = H	0.0 *^d^*	(5.45 ± 0.13) × 10^−3^	14.9	−14.2
	10.0	(1.49 ± 0.02) × 10^−2^		
	25.0 ^*c*,*e*^	5.95 × 10^−2^		
**1**, Z = Cl	5.1	(6.67 ± 0.07) × 10^−4^	15.5	−17.2
	25.0 *^f^*	(4.65 ± 0.01) × 10^−3^		
	25.0 ^*f,g*^	(4.54 ± 0.07) × 10^−3^		
**1**, Z = NO_2_	25.0 *^h^*	(1.77 ± 0.08) × 10^−6^		

a Determined conductimetrically in duplicate except where state otherwise; errors are average deviations;

b Structures are given in Figure 1;

c Calculated from data at other temperatures;

d Triplicate measurements of rate constant;

e Additional single measurements in different solvent batches gave *k* = (5.98 ± 0.04) × 10^−2^ s^−1^ and (5.54 ± 0.04) × 10^−2^ s^−1^, and a value of (6.28 ± 0.07) × 10^−2^ was determined by G. E. Carter [13];

f Literature value = (3.21 ± 0.08) × 10^−3^ [7], determined titrimetrically;

g Determined in duplicate by UV monitoring;

h Literature value [7].

**Table 2.** Rate constants (*k*/s^−1^) and activation parameters for solvolyses of *p*-methoxy benzoyl chloride (**1**, Z = OMe) in formic acid *^a^*.

**Substrate *^b^***	**Temperature/°**C	***k*/s^−1^**	**ΔH^≠^/kcal mol^−1^**	**ΔS^≠^/cal mol^−1^ K^−1^**
**1**, Z = OMe	5.0 *^d^*	(2.53 ± 0.21) × 10^−1^	18.0	3.4
	10.0	(4.57 ± 0.10) × 10^−1^		
	25.0 *^c^*	2.4		

a–d As for Table 1.

**Table 3.** Rate constants (*k*/s^−1^) for acetolysis and formolysis of *p*-substituted benzoyl chlorides (**1**) at 25 °C *^a^*.

**Substrate**	**Acetic acid**	**Note *^b^***	**Formic acid**	**Note *^b^***
**1**, Z = OMe	(1.23 ± 0.11) × 10^−4^	UV ^*c*,*d*^	2.4	Table 2
	(1.18 ± 0.07) × 10^−4^	Titr		
**1**, Z = Me	3.98 × 10^−5^	[15]	(1.53 ± 0.03) × 10^−1^	Cond *^c^*
**1**, Z = H	(6.7 ± 0.3) × 10^−6^	Titr *^e^*	(2.11 ± 0.14) × 10^−2^	Cond ^*f*,*g*^
**1**, Z = F			(7.3 ± 0.2) × 10^−3^	Cond *^c^*
**1**, Z = Cl	(1.42 ± 0.12) × 10^−6^	Titr	(2.74 ± 0.16) × 10^−3^	Cond ^*h*,*i*^
			(1.71 ± 0.05) × 10^−3^	UV ^*h*,*j*^
**1**, Z = NO_2_	1.05 × 10^−6^	[15]	2.09 × 10^−5^	[15]

a Determined in duplicate, except where state otherwise; errors shown are average deviations;

b UV refers to continuous spectrophotometric monitoring; Titr refers to titrimetric analysis of aliquots at set times; [#] is a reference number;

c Triplicate measurement of *k*;

d Added 2% acetic anhydride to remove traces of water; higher values (2.1 ± 0.1 × 10^−4^) were obtained in the absence of anhydride;

e Literature 1.05 × 10^−5^ [15];

f Five measurements of *k*;

g A single measurement of *k* by UV monitoring gave *k* = (1.66 ± 0.03) × 10^−2^ s^−1^; previously estimated [13] from data [16] at 9 °C, *k* = 2.0 × 10^−2^;

h Six measurements of *k*;

i Additional measurements in a solution containing pre-reacted 10^−2^ M acid chloride (giving HCl) gave a slightly higher result of *k* = (3.2 ± 0.1) × 10^−3^ s^−1^;

j An additional measurement in the presence of 2% acetic anhydride gave *k* = 1.47 × 10^−3^ s^−1^.

**Table 4.** Calculated energies, stabilisation energies (SE, Equation 2) and heterolytic bond dissociation energies (HBDE) for benzoyl chlorides *^a^*.

**Substrate**	**E(ArCOCl)/Hartrees *^b^***	**E(ArCO^+^)/Hartrees *^b^***	**Stabilisation energies/kcal mol^−1^**			**HBDE/kcal mol^−1^**
			**HF/6–31G(d)**	**B3LYP/6–31G(d)**	**6–311G(d,p)**	
**1**, Z = H	−802.34371 *^c^*	−342.59639 *^c^*	0.0 *^d^*	0.0 *^d^*	0.0 *^d^*	150.1 *^e^*
**1**, Z = F	−901.19505	−441.44325	2.8	1.6	2.07 *^f^*	152.3
**1**, Z = Cl	−1261.24202 *^c^*	−801.48796 *^c^*	4.2	2.3	2.11 *^f^*	153.3
**1**, Z = NO_2_	−1005.80911 *^g^*	−546.03707 *^g^*	15.5		12.05 *^f^*	163.9
**1**, Z = NMe_2_	−935.42722	−475.70965	−18.7*^h^*	−20.2*^h^*		130.7*^h^*
**1**, Z = OMe	−916.22720 *^c^*	−456.49301 *^c^*	−8.2	−9.6	−9.54 *^f^*	141.2
**1**, Z = Me	−841.38219 *^g^*	−381.64104 *^g^*	−3.9		−4.54 *^f^*	145.9
2,6-diMe (**3**)	−880.40699 *^c^*	−420.68210 *^c^*	−14.1 *^h^*	−14.5 *^h^*		135.8 *^h^*
2,6-diCl (**4**)	−1720.12086	−1260.37391	−0.2	−2.7	−1.8	148.9
2,4-diCl (**5**)	−1720.12652	−1260.37638	1.8	−0.1		151.0
3,4-diCl	−1720.13317	−1260.37130	9.1	6.0		157.6
3,5-diCl	−1720.13634	−1260.37021	11.8	9.5		160.8
2,6-diF (**6**)	−1000.03037	−540.28117	1.2	0.0		150.7
3-OMe *^i^*	−916.22245	−456.47692	−1.1	−3.3		147.9

a Data either from literature quoted, or calculated using Gaussian 03; HBDEs are obtained by adding the average of two stabilisation energies to the value of 150.1;

b These values refer to HF/6–31G(d) calculations;

c In agreement with published values [18];

d By definition;

e Calculated value from reference [24];

f Includes a small correction for differences in zero point energies; data from reference [26];

g Reference [18];

h Reference [24];

i 3-Methoxybenzoyl chloride.

**Table 5.** Rate constants (*k*) and calculations of log *k* at 25 °C for benzoyl chlorides in 97% w/w hexafluoroisopropanol-water (97H).

**Substrate *^a^***	***k*/s^−1^ (55 °C) *^b^***	***k*/s^−1^ (25 °C)**	**log *k*_obs_*^c^***	**log *k*_calc_*^d^***	**Δlog *k**^e^***
3,4-dichloro	1.40 × 10^−3^	1.12 × 10^−4^	−3.92	−3.7	0.2
3,5-dichloro	1.35 × 10^−4^	7.43 × 10^−6^*^b^*	−5.13	−4.7	0.4
2,6-diMe (**3**)			3.9 *^f^*	3.2	−0.7
2,6-dichoro (**4**)	5.62 × 10^−2^	5.6 × 10^−3^*^g^*	−2.25	−0.95	1.3
2,4-dichloro (**5**)	1.51 × 10^−3^	1.5 × 10^−4^*^g^*	−3.8	−1.6	2.2
2,6-difluoro (**6**)	3.46 × 10^−3^*^h^*	3.5 × 10^−4^*^g^*	−3.45	−1.5	1.95

a See Figure 1;

b Data from reference [6];

c At 25 °C;

d From Equation 3;

e Δlog k = log *k*_calc_ *−* log *k*_obs_;

f By extrapolation of a correlation (Figure 2 of reference [5]) of log *k* for **3** *vs.* log *k* for **1**, Z = OMe;

g Estimated by dividing log *k* at 55 °C by 10;

h Data from reference [8].
